# Winner Winner Turkey Dinner! An Empirical Approach to Measuring Palatability and Satisfaction with Emergency Department Turkey Sandwiches

**Published:** 2022-04-01

**Authors:** D Toomey, CE Goldfine, TB Erickson, PR Chai

**Affiliations:** 1 Department of Emergency Medicine, Brigham and Women’s Hospital, USA; 2 Harvard Humanitarian Institute, USA; 3 Department of Psychosocial Oncology and Palliative Care, Dana Farber Cancer Institute, USA; 4 The Koch Institute for Integrated Cancer Research, Massachusetts Institute of Technology, USA; 5 The Fenway Institute, USA

## Abstract

**Introduction:**

Despite their popularity in many EDs, little is known regarding perceptions of turkey sandwiches among patients. Given the importance of turkey sandwiches as a form of nutrition provided in EDs, we sought to quantify the composition and taste of ED turkey sandwiches through a quantitative assessment of turkey sandwiches by ED staff.

**Methods:**

This was a blinded observational study performed at a tertiary, urban academic medical center in Boston, MA. We collected ED turkey sandwiches up to 48 hours prior to study days from 4 emergency departments in the Boston area (2 community hospitals and 2 academic medical centers). We enrolled ED physicians, nurses, physician assistants, and staff who were exposed to four sandwiches in a random fashion. Participants were asked to assess sandwiches on a Likert scale of 1 to 5 on a variety of factors including, nutritional value “goodness”, smell “olfactory”, texture “bite”, ingredient distribution “balance”, appearance “look”, and flavor “edibility” to produce a composite score for sandwich quality (GOBBLE score). Next, participants were asked standardized questions surrounding suitability for consumption and nutrition on a 10 point Likert scale. We calculated mean scores and measured differences using t-tests.

**Results:**

We enrolled 22 participants over the study period. Twenty-one participants completed all measures. GOBBLE scores were calculated and averaged for sandwiches. A One-way ANOVA test was performed to measure statistically significant differences between mean GOBBLE scores (p<0.05) with a post hoc Tukey HSD procedure used to assess for statistically significant difference for pairwise comparison. A significant difference (p=0.009) was noted between the 4 sandwiches being compared, with a single site outperforming the others. Aggregating for academic and community sites demonstrated no statistically significant difference (p=0.08). Sandwiches in general were not considered healthy or palatable by study subjects.

**Conclusion:**

Despite out-performance by a single ED, there is no significant difference in the quality of sandwiches provided to patients in EDs between academic and community hospitals. ED turkey sandwiches do not appear to be food items that are viewed as healthy or recommended to patients by ED staff who participated in this study.

## Introduction

The Emergency Department (ED) is an important setting in which providing nutrition may be critical in both clinical assessment and in shaping overall hospital experience and satisfaction for both patients and their families [1]. The purpose of providing food items to patients is to provide nutrition to aid in the process of healing and recovery and to prevent further comorbidity associated with low caloric intake. Several studies have additionally demonstrated that provision of food services customized to patient needs also provides an opportunity for patient nutritional education, particularly in the setting of underlying comorbidity or acute illness [2,3].

Given the importance of nutrition in the ED, the quality of food items therefore, is of particular importance. Multiple studies have demonstrated significant rates of food insecurity among both pediatric and adult ED patients as a whole [4–8], with further research correlating timing of ED visits with periods of acute food insecurity [9,10]. Food quality has also been demonstrated to serve as a significant factor in ED specific patient satisfaction for both food secure and food insecure populations [11,13]. The diversity of the patient population in terms of personal and religious preferences as well as dietary restrictions including food allergies, present challenges in the development of ‘uniform’ ED food selections. For many EDs in the United States, the turkey sandwich has become a staple food source made readily available to patients. While there is a lack of formal research recognizing the ubiquity of turkey sandwiches across EDs, colloquial knowledge suggests that turkey sandwiches are one of the most commonly provided food items in EDs across the country [14,15]. Several theories have been forwarded as to why this is the case, including overall protein content, low sodium content, and the fact that turkey meat is typically both kosher and halal thereby serving as a universally acceptable food source for the majority of non vegetarians in the ED setting.

Despite the ubiquity of turkey sandwiches in EDs and the increasing emphasis on food quality in US hospital systems as a whole, there is a lack of data examining the overall quality of turkey sandwiches provided to patients in the clinical setting. The aims of this investigation were to 1) develop a scoring system by which turkey sandwiches could be assessed across multiple hospital settings, 2) to assess basic quality metrics for turkey sandwiches served at individual hospital study sites, and 3) to assess for differences in overall quality between turkey sandwiches provided at community EDs compared to academic medical centers.

## Methods

**Figure 1:**
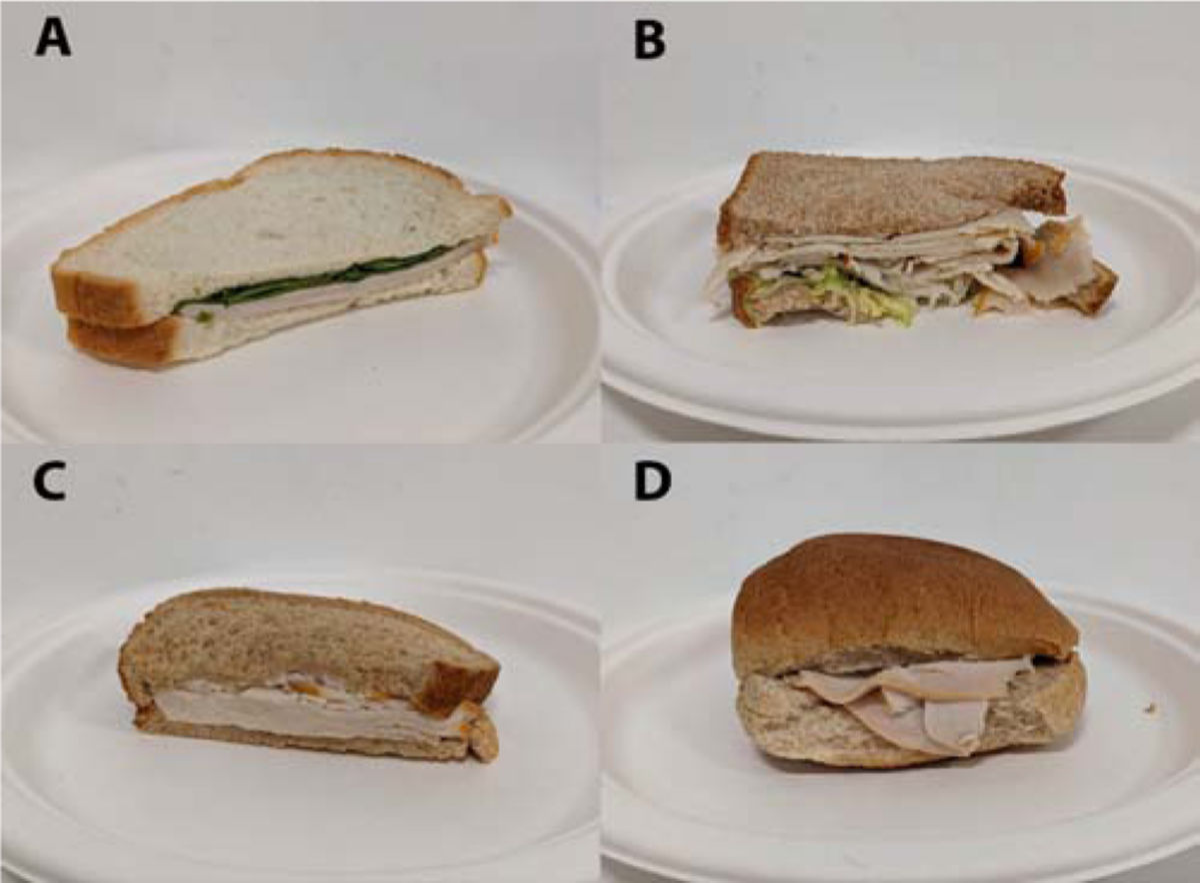
Selected turkey sandwiches used in the study. **Reference:** A, D = Community; B, C = Academic

This was a blinded observational study performed at a tertiary, urban academic medical center in Boston, MA. Study procedures were approved by the Massachusetts General Brigham Institutional Review Board. We selected 2 community EDs and 2 urban academic tertiary care center EDs within our hospital network for study sites. The average annual patient volume for each study site was 45,000 for community EDs and 80,000 for academic centers. Twenty four to 48 h prior to designated study days, staff collected ED turkey sandwiches from 4 EDs in the Boston area and immediately stored them in a designated study refrigeration unit at 4 degrees centigrade (Figure 1). All sandwiches were examined to ensure that no food items would be used past their designated expiration date as noted on item packaging, and that the packaging was not violated prior to use. Condiments were often provided along selected food items within the package, but for the purpose of uniform presentation, condiments were not added to sandwiches included in the study. If the sandwiches already contained vegetable matter such as lettuce or tomatoes, these sandwich items were left in place and unaltered. Sandwiches were removed from packaging and presented on clean paper plates to study subjects during study sessions. We enrolled participants who were physicians, nurses, physician assistants, and ancillary staff working in the ED of the main study site to grade sandwiches in a randomized, blinded fashion. In each session, participants were presented with samples of turkey sandwiches from each study site in a similar blinded and randomized manner. We next asked participants to complete a quantitative assessment measuring sandwiches in a variety of factors including nutritional value “goodness”, smell “olfactory”, texture “bite”, Ingredient distribution “balance”, appearance “look”, and flavor “edibility” to produce a composite score for sandwich quality (G.O.B.B.L.E. score). Questions comprising the GOBBLE score were asked on a one to five Likert scale. Next, participants were asked questions surrounding suitability for consumption and nutrition on a 10 point Likert scale. The quantitative assessment was designed by the study staff. We next piloted the survey among study team members to ensure completeness and comprehension. We deployed the final survey among study participants. This investigation was reviewed and approved by the Mass General Brigham Institutional Review Board.

### Data Analysis

We calculated basic descriptive statistics for demographic values. We then calculated a mean score for each category of the GOBBLE score and a mean composite GOBBLE score (Table 1). Next, we measured the difference in GOBBLE score between individual sites using a t-test. Given that our a-priori hypothesis was that there would be a significant difference in GOBBLE score between academic and community emergency department turkey sandwiches, we grouped both academic and community ED scores together and measured the difference between GOBBLE scores in these two groups. Finally, a post-hoc Tukey HSD procedure using GOBBLE scores was performed in a pairwise fashion between study sites to assess for individual outperformance by a single sandwich.

## Results

We enrolled 22 participants over the study period. Twenty-one participants completed all measures. GOBBLE scores were calculated and averaged for sandwiches (Table 2).

One-way ANOVA test was performed to measure statistically significant differences between mean GOBBLE scores (p<0.05) with a post hoc Tukey HSD procedure used to assess for statistically significant difference for pairwise comparison. Using this, a significant difference (p=0.009) was noted between the 4 sandwiches being compared, with post hoc Tukey HSD demonstrating significant differences in pairwise comparisons between sandwiches A:C (p=0.14) and B:C (p=0.02). Aggregated for academic and community sites (comparing A+D, C+B), demonstrated no statistically significant difference (p=0.08). Significant differences were noted between sandwiches for ‘Would you eat’ (p=0.048) and ‘Would you recommend’ (p=0.03). No significant pairwise difference was noted for ‘Would you eat’ groups, but for ‘Would you recommend’ a significant pairwise difference was noted between A:C (p=0.004) and B:C (p=0.018). No significant difference was noted for responses to ‘Is this sandwich healthy’ (p=0.311).

## Discussion

Of all the sources of food that are commonly available to United States ED patients, the turkey sandwich is ubiquitous and universally recognized by patients and staff. While empirical data is lacking, there is anecdotal evidence suggesting that ED staff negatively perceive the quality of turkey sandwiches served in the ED. To our knowledge, this investigation is the first study to demonstrate that ED staff poorly score the palatability and presentation of ED turkey sandwiches, and have a negative attitude towards their health benefits. Additionally, contrary to popular belief, we found no significant difference in perceived quality and palatability of turkey sandwiches between academic and community EDs.

This investigation demonstrated a negative perception of turkey sandwiches by ED staff. Composite GOBBLE scores were a mean of 17.12, only slightly more than half of the total possible points on the scale. Additionally, each subscale demonstrated a negative perception of turkey sandwiches through all subscales of the GOBBLE score. It is unclear what led to these perceptions among study participants. We anticipate there may be implicit biases around the quality of turkey as “hospital food” that is distributed to ED patients that occur during training of ED staff. Despite these biases, it is important to note that along with poor ratings of the quality of turkey sandwiches, ED staff reported that they would not recommend sandwiches the patients. This data suggests that while turkey sandwiches function as a ubiquitous source of food in emergency departments, there is room for improvement in both the physical composition of sandwiches to make them appealing as well as the content of sandwiches to improve their potential nutritional value.

While an ED turkey sandwich is unlikely to ever be comparable to gourmet sandwiches at restaurants, we noted that the lack of both tomatoes and condiments were universal through academic and community samples. We suspect these measures may have been deliberately taken in order to prolong the shelf life or decrease the cost of stocking and providing sandwiches in the ED. Despite this, we anticipate GOBBLE scores may have been higher with the incorporation of these options in ED turkey sandwiches. Future development of ED turkey sandwiches may consider preliminary testing among ED staff as they may be individuals who would be offering this food source to patients.

Interestingly, we also found that there was no difference in the perceived quality of turkey sandwiches when comparing academic to community emergency departments. Contrary to urban legend where the quality of food products at community EDs is believed to be superior to that at urban, academic EDs, our investigation demonstrated no statistical significance in GOBBLE scores which included taste of sandwiches. Additionally, participants reported that they would be unwilling to recommend either sandwich. This data demonstrates that in our sampling of EDs, there is no difference in the quality of turkey sandwiches provided at community versus academic EDs and efforts at improving food quality may benefit both environments.

Overall, this study demonstrates that despite out performance by a single ED, turkey sandwiches are rated poorly among ED staff and not recommended to patients. Additionally, this premise is consistent when community EDs are compared to academic EDs. Our investigation suggests that innovation in provision of food in the ED may be a potential avenue to improve nutrition and patient satisfaction in the ED.

### Limitations

This investigation had several limitations. First, we surveyed a small number of EDs that provided turkey sandwiches within a large city on the East Coast. Our results may therefore not be generalizable across the United States. Second, we focused on EDs in our hospital system and therefore did not address hospital system level differences in turkey sandwiches. Third, we presented sandwiches without their native packaging. It is possible that aspects of the GOBBLE score related to presentation and even palatability may have been altered if sandwiches were presented in their original packaging. Fourth, we did not ask ED patients to opine on the quality of turkey sandwiches. Patient perspectives may vary from ED staff member perspectives. Finally, we utilized an unvalidated scoring system to understand palatability of turkey sandwiches. Future research may need to focus on validation of the GOBBLE score.

## Conclusion

Despite being a ubiquitous food offering in emergency departments across the country, turkey sandwiches served at both academic and community EDs were not perceived as palatable, nutritious, or appealing by emergency medicine staff. Further investigation is needed as to how quality assurance measures can be implemented to improve food product options for ED patient populations.

## Figures and Tables

**Table 1:** GOBBLE subscales and composite scores for turkey sandwiches.

Sandwich	Goodness	Olfactory	Bite	Balance	Look	Edibility	Mean
A (community)	2.57	2.86	2.52	2.57	2.61	2.38	2.58
B (academic)	2.61	2.76	2.76	2.52	2.42	2.67	2.62
C (academic)	3.24	3.24	3.43	3.43	3.52	3.53	3.4
D (community)	2.67	3.14	3.24	2.71	3.04	2.9	2.95

**Table 2:** Averaged composite GOBBLE scores and averaged responses for suitability for consumption for turkey sandwiches.

Sandwich	GOBBLE score (6–30)	Would you eat? (1–10)	Would you recommend? (1–10)	Is this sandwich healthy? (1–10)
A (community)	15.4	4.33	4	4.05
B (academic)	15.63	4.52	4.29	3.76
C (academic)	19.86	6.14	6.09	4.91
D (community)	17.59	5	4.8	4.2
Mean	17.12	4.99	4.79	4.23
